# Evaluation of the Use of Pyrolysis Oil from Municipal Solid Waste in the Germination and Development of Lettuce (Lactuca sativa), Biochemical Properties, and Level of Contaminants

**DOI:** 10.1021/acsomega.4c10325

**Published:** 2025-06-12

**Authors:** Rafaela Meneguzzo, Wendel Paulo Silvestre, Marcelo Godinho, Gabriel Fernandes Pauletti

**Affiliations:** Postgraduate Program in Process Engineering and Technologies (PGEPROTEC), 58802 University of Caxias do Sul, Caxias do Sul 95070-560, Rio Grande do Sul, Brazil

## Abstract

Pyrolysis oil, one of the products of the pyrolysis process of different wastes, due to its diverse chemical composition, may have biological activity of interest for agricultural areas. However, the safety of using these products must be adequately evaluated to verify the applicability of pyrolysis oil from municipal solid waste (MSW) on agricultural crops. This study carried out the quantitative chemical characterization of MSW pyrolysis oil and evaluated the germination inhibition potential of this material on lettuce seeds in a laboratory test carried out at the concentrations of zero (control), 0.1%, 0.5%, 1.0%, 1.5%, and 2.0% v/v. Moreover, the pyrolysis oil was sprayed on lettuce plants, with a weekly application of pyrolysis oil for 4 weeks, at concentrations of zero (control), 25%, 50%, 75%, and 100% v/v, and the plants’ growth was assessed. The main constituents identified in MSW pyrolysis oil were hydrocarbons, phenols, esters, amides, and organic acids. The pyrolysis oil showed satisfactory germination control with increasing concentrations, did not cause toxicity effects in plants, and did not cause significant changes in the levels of phenolic compounds and flavonoids in plant tissue. The levels of heavy metals remained within the maximum limits established by legislation for fresh food. The pyrolysis oil from municipal solid waste showed promising results in the exploratory tests, demonstrating its suitability for studies and its use within the agricultural sector.

## Graphical abstract

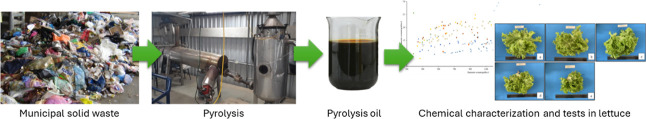

## 1 Introduction

Lettuce (Lactuca sativa L.) is one of the most consumed leafy vegetables worldwide, with a global production of 27.1 million tonnes, whose primary producers are China, the United States, and India.^1^ In Brazil, its commercialization generates around BRL 8 billion in retail, with an average production of 1.5 million tons per year.^2^ There are more than 840 registered varieties of the crop, bringing diversity to the Brazilian table.^3^ Because it is economically viable for consumers and is produced throughout the year, it is a crop of great importance for Brazilian and global agriculture.^4^

The use of products obtained through the pyrolysis process, a thermal degradation process in anoxic (without oxygen) conditions, has been recurrent in research in different areas, with these products being used as herbicides, fungicides, insecticides, and fertilizers, seeking to verify their efficiency for control and evaluate their toxicity on agricultural crops, animals, and humans.

Given the environmental problem caused by the disposal of municipal solid waste (MSW), pyrolysis is seen as a substitute alternative for the disposal of this material in landfills and dumps, generating three byproducts: biochar (solid fraction), pyrolysis oil (liquid fraction), and noncondensable gases (gaseous fraction), which have been studied to evaluate their agricultural suitability, both for food and industrial crops.

Food safety is a great concern, and agricultural practices aim to provide the population with healthy, risk-free food. In this sense, extensive national and international legislation regarding the safe amount of contaminants in food exists. Bioactive compounds, including phenolic and flavonoid compounds, indicate beneficial effects due to antioxidant and antiproliferative activity, which are directly linked to a reduction in the development of atherosclerosis and cancer.^5^ At an agronomic level, the higher the content of bioactive compounds present in the plant, the greater its resistance to sources of stress, such as drought, excessive sunlight, and attacks by predators and pathogens, for example.

The use of pyrolysis oil in agriculture is still preliminary, with the intent to evaluate the effects it causes on plants since, in other studies, the chemical composition found resembles products already used in the daily production of some crops.^6^

The main advantage of using MSW pyrolysis oil is based on a waste management standpoint. MSW, a widespread problem worldwide, can be transformed into products with added value and possibly used in other critical areas, such as agriculture and soil remediation. As a disadvantage, the variable composition of MSW can generate pyrolysis oils with quite distinct compositions that may render them unusable for some applications.^7,8^ The characterization of pyrolysis oils is important for defining project parameters, developing commercial plants, scaling up, and making decisions regarding producing and using this kind of byproduct.^9,10^

Although obtained from an uncommon source (MSW), MSW pyrolysis oils have a composition that resembles that of biomass and other types of agroindustrial wastes. However, the exact composition depends on the pyrolysis system, temperature conditions, and plastic content in MSW.^11^ This means that MSW pyrolysis oil may have a performance similar to that of pyrolysis oils obtained from biomass, although this must be assessed in more depth.^12^

Velghe et al. and Hasan et al. commented that, depending on process conditions, MSW pyrolysis oil is desirable for fuel and chemical feedstock use.^11,12^ However, de Souza et al. highlighted that MSW pyrolysis oil may be an important source of agrochemicals and a feedstock for use in agriculture.^6^ Nevertheless, due to the small number of studies that address this issue, further investigation is needed to assess the potential and safety of using such a material in agricultural crops.

Given the above, the present study aimed to evaluate the effects of the application of pyrolysis oil from urban solid waste on the development of lettuce plants, from seedlings to grown plants, seeking to verify the suitability of this product for agricultural use, determining whether there was an increase in the levels of heavy metals, in addition to evaluating the levels of nutrients and bioactive compounds.

## 2 Materials and Methods

The pyrolysis oil used in this study was obtained through the slow pyrolysis of municipal solid waste. The material used in the pyrolysis process was the organic material that cannot be recycled (i.e., all remaining material after removing glass, metals, and recyclable plastics). Pyrolysis was conducted in the Experimental Area and School Farm of the University of Caxias do Sul. An inox Auger-type reactor (endless screw) was used, with a final pyrolysis temperature of 450 °C, a heating rate of 5 °C min^–1^, and a residence time of 90 min. Five different pyrolysis runs were carried out using the sorted MSW waste. The oil was used *in natura* without filtration or subsequent purification.

For the germination test, lettuce seeds (L. sativa L.), commercially known as *Crespa Grand Rapids*, Isla brand, without pretreatment and not pelleted, with 99.7% purity and 96.0% germination, according to the manufacturer’s data, were used. The test followed the methodology described in the Rules for Seed Analysis—RAS.^3^ The seeds were distributed on hydrated germ test paper with 12.5 mL of solution, with the following treatments: control (water only), 0.1%, 0.5%, 1.0%, 1.5%, and 2.0% v/v of pyrolysis oil in distilled water, generating a true solution (only the supernatant of the mixture was used). These concentrations were used because they are usually used in tests of this nature since the study is preliminary.

The evaluations were carried out 24, 48, and 72 h after germination of the control (radicle emission), and the observed parameters were the germination speed index (GSI), calculated according to eq 1, proposed by Carvalho and Carvalho,^13^ and the germination percentage.

1 $$GSI=\sum \frac{n_{i}}{t_{i}}$$

where GSI = germination speed index; *n*_*i*_ = number of seeds that germinated in time ″*i*″; *t*_*i*_ = time for seeds to germinate after test installation.

The seed was considered germinated when the radicle had a length equal to or greater than 2.0 mm.

For chemical characterization, two-dimensional chromatographic analysis coupled with mass spectrometry (GC × GC/MS) was performed on a LECO Pegasus 4D equipment (LECO, Saint Joseph, MI, USA). One microliter of the bio-oil sample was analyzed with a flow division of five, maintaining the injector temperature at 300 °C. The concentrations of bio-oil in dichloromethane and the standard mixture used were 100 mL·L^–1^ and 50 mg·L^–1^, respectively. Helium gas (purity greater than 99.999%) was used as the carrier gas at a flow rate of 1.4 mL·min^–1^.

The column sets used for the analyses were DB-5 in the first dimension, with dimensions of 30 m × 0.25 mm × 0.25 μm, and DB-17 in the second dimension, with dimensions of 0.75 m × 0.25 mm × 0.25 μm. The mass spectra were acquired in the 35–600 a.m.u range with a data acquisition rate of 200 Hz and 70 eV (electronic impact), maintaining the transfer line at 300 °C and the ion source at 250 °C.

The modulator chiller was set at −80 °C, and the modulation period used was 3.0 s (0.9 s for hot jets and 0.6 s for cold jets). For the GC × GC conditions, initial oven temperatures of 65 °C (1 min) to 200 °C (0.5 min) at a heating rate of 7.0 °C·min^–1^ and from 200 to 300 °C (1 min) at a heating rate of 10C·min^–1^ were used, maintaining the difference between the temperatures of the primary and secondary ovens at 5 °C throughout the analysis. Data processing was performed by Chroma TOF software version 451.6 through an integrated spectral deconvolution tool, capable of differentiating coeluted mass spectra into chromatographic peaks.

For the plant development test, seedlings produced at the University of Caxias do Sul from the same batch were used for the germination test. The seedlings were grown in a greenhouse with controlled temperature, precipitation, and humidity conditions and, after reaching the size for transplanting, were placed in 3.8 L tubes and grown in a Carolina Soil substrate with fertigation.

Weekly, the plants were sprayed (aspersion) with the pyrolysis oil solution in tap water, using a manual sprayer, with the control being without the use of pyrolysis oil, and concentrations of zero (control), 25%, 50%, 75%, and 100% v/v of pyrolysis oil, until the point of runoff. Likewise, the concentrations used come from other similar studies.

After 4 weeks of cultivation and pyrolysis oil application, the plants were collected, and the fresh mass was determined. The samples were subdivided into two parts. One part was used to determine the nutrient content in the plant tissue, according to the method described by Malavolta et al.^14^ The other part was used to analyze the levels of heavy metals (Al, As, Ba, Cd, Co, Cr, Hg, Mo, Ni, Pb, and Sn) according to the AOAC 999.10 and AOAC 2013.06 methods in the plant tissue.^15^

The content of phenolic compounds and flavonoids was determined by analyzing the extract after extraction with 96% v/v ethanol in 5 g of sample and 50 mL of ethanol. The levels of phenolic compounds were determined by the Folin–Ciocalteu method, according to the procedure proposed by Pereira et al.^16^ The total flavonoids were determined by the aluminum chloride spectrometric method, according to the procedure proposed by Matic et al.^17^

Lettuce was chosen to carry out the experiment because it is a standard crop for germination and phytotoxicity tests as it is a sensitive species with a short cycle. The experimental design used was randomized, where the factor evaluated was the different concentrations of pyrolysis oil sprayed weekly. Each treatment consisted of ten plants. The variable analyzed was the fresh weight of the plants. The data were subjected to analysis of variance (ANOVA), followed by Tukey’s multiple mean comparison test at a 5% error probability, using the Agroestat software (Brazil).

## 3 Results and Discussion

The chemical characterization of MSW pyrolysis oil was performed at a qualitative level, which was characterized by heterogeneity and the presence of several chemical functions. The approximate proportions of the detected peaks in terms of chemical classes are listed in Table 1.

**Table 1:** Proportion (%) of Chromatographic Peaks in Chemical Classes, Detected via GC × GC/MS, for Municipal Solid Waste (MSW) Pyrolysis Oil

chemical class	occurrence percentage^a^
acids	6.18
alcohols	4.49
aldehydes	3.93
amides	8.99
amines	3.93
ketones	2.81
epoxides	0.56
esters	17.42
ethers	1.12
phenols	13.48
hydrocarbons	34.27
nitriles	2.25
sulfones	0.56

a The occurrence percentage is calculated based on the relative peak area of each compound, grouped in chemical classes, and represents a rough estimate of the composition of the pyrolysis oil analyzed.

The higher proportion of peaks characterized as phenols in MSW pyrolysis oil may be associated with plastic derivatives (polymers), which commonly have aromatic and/or phenolic groups generated in the pyrolytic process.^18–20^

Table 2 shows the results of the germination percentage and the germination speed index (GSI) of lettuce seeds when exposed to different concentrations of MSW pyrolysis oil.

**Table 2:** Germination Percentage and Germination Speed Index (GSI) of Lettuce Seeds Subjected to Different Concentrations of Pyrolysis Oil from Municipal Solid Waste in a Germination Test^a^

concentration	germination (%)	GSI
control	100a	25.0a
0.1% v/v	99.0a	24.75a
0.5% v/v	95.0a	23.25a
1.0% v/v	82.0b	17.77b
1.5% v/v	62.0c	12.96c
2.0% v/v	47.0d	10.65c
coefficient of variation	7.57	7.14

a Means followed by the same letter in the column do not differ statistically according to the Tukey test at a 5% error probability.

It is possible to verify that the increase in the concentration of MSW pyrolysis oil reduces the germination potential of the lettuce seeds and delays the germination time satisfactorily, when this is intended. Higher concentrations tend to inhibit development and delay the germination process entirely.

Germination is a widely used parameter in evaluating the presence of allelopathy due to its speed and ease of execution. However, it is essential to highlight that the germination process is less sensitive to the effects of allelopathy compared to the subsequent growth and development of plants. As highlighted by Cândido et al.,^21^ allelochemical effects can arise after germination, resulting in anomalous seedlings, which may present deficiencies in both the aerial part and the root, and it is possible that the seeds that germinated present some damage later in their development.

In a study carried out by Bonow,^22^ with oil from the pyrolysis of rice husk, it was possible to verify a similar result, where there was inhibition of lettuce germination from a concentration of 1.0% v/v. Another similar study evaluated *Carum carvi* seeds in contact with wood pyrolysis oil, with no germination occurring in concentrations close to 20% v/v, with the presence of organic acids being discussed as being responsible for inhibiting germination,^23^ a fact that can be observed in the chemical characterization carried out, where there is the presence of acids in a high percentage (Table 1).

It is important to observe that pyrolysis oils, especially from lignocellulosic biomasses, are characterized by the presence of organic acids, which might have an acidification effect, which can cause phytotoxicity and enhance the toxicity of other compounds present in the pyrolysis oil. Although MSW has a highly variable composition, lignocellulosic materials, such as papers, may compose an important fraction of such wastes, contributing to the generation of compounds that may be toxic.^21,22^

In a subsequent study, lettuce was sown and kept in a greenhouse until it reached the ideal size for transplanting into tubes. Treatments were applied weekly, and after the end of the cycle (30 days), the lettuce plants treated with different concentrations of pyrolysis oil were harvested and weighed on a precision scale. The fresh weight of the plants is compiled in Table 3.

**Table 3:** Fresh Mass of Lettuce Plants Subjected to Different Doses of Pyrolysis Oil from Municipal Solid Waste^a^

concentration	fresh mass (g)
control (zero)	177.2a
25%	178.4a
50%	164.4ab
75%	127.2ab
100%	103.0b
coefficient of variation (%)	26.10

a Means followed by the same letter in the column do not differ statistically by Tukey’s test at a 5% error probability.

Statistically, the plants showed differences in fresh mass only at the highest concentration of MSW pyrolysis oil (100% v/v), with lower fresh mass in this treatment. Fedelli^24^ applied wood pyrolysis oil to lettuce seedlings, following a methodology like that used in this work, using concentrations of 0.25% and 0.50% v/v, and observed an increase in the biomass of the treated plants.

Blueberry plants, in a trial carried out by Zhang et al.,^25^ were subjected to treatment with pyrolysis oil from wood waste in a proportion of 0.2% v/v and showed an increase in yield and number of fruits, indicating that the product has potential for use since it has been showing positive results, whether in horticulture or fruit growing.

The addition of 10% v/v of pyrolysis oil in combination with fertilizer formulations already available on the market provided an increase in the weight and size of watermelon fruits without causing damage to the fruits and, consequently, increasing the final productivity of the crop in a study by Zulkarami et al.^26^

Polthanee et al.^27^ used eucalyptus bio-oil to produce upland rice and cattle manure. They observed an increase in productivity parameters, grain yield, plant height, and number of panicles, showing that the liquid fraction of biomass pyrolysis can be used as an enhancer of products already known in agriculture, able to add value to them.

Pyrolysis oil was also tested in rapeseed plants by Zhu et al.,^28^ where they observed an increase in the dry and fresh weight of seeds, plant size, and total number of leaves. It was also observed that the oil delayed leaf senescence, facilitating the accumulation of dry matter and transporting photoassimilates to the fruiting organs, consequently increasing the seed yield in terms of mass.

Toxicologically, the lettuce plants did not show symptoms of phytotoxicity (Figure 1) since they were treated with high concentrations of bio-oil, a fact of great importance since the crop used in the work is quite sensitive and commonly used in toxicity tests, whether by direct contact or by root absorption.

**Figure 1:**
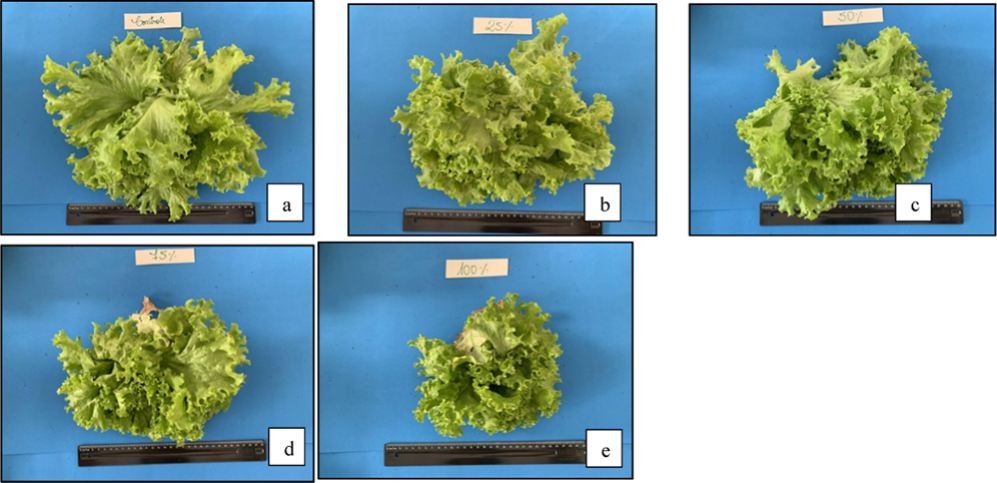
Visual aspect of the growth of lettuce plants after weekly spraying with different concentrations of pyrolysis oil from municipal solid waste for 30 days: (a) control, (b) 25%, (c) 50%, (d) 75%, and (e) 100% v/v pyrolysis oil.

Toxic effects may arise from different mechanisms that depend on the administration route, such as root absorption or direct contact with the roots. Such mechanisms may be toxicity from components of the pyrolysis oil or potentiation of other effects, such as the production of free radicals and damage to cell structures.^18,19^

While the seedlings showed a growth reduction (Table 2), the plants treated by aspersion (direct contact) did not demonstrate any important toxicity symptoms, except for smaller size and mass for the plants treated with 100% MSW pyrolysis oil. Although statistically significant, the mass of the plants treated with 100% MSW pyrolysis oil has not differed from those treated with 50% and 75% pyrolysis oil (Table 3).

Among the experiment’s objectives, in addition to evaluating the development of signs of phytotoxicity in plants, it was also sought to assess the nutrient content in plant tissue, whose results are presented in Table 4.

**Table 4:** Results of Leaf Tissue Analysis of Lettuce Plants Subjected to Different Concentrations of Pyrolysis Oil from Municipal Solid Waste

treat	N	P	K	Ca	Mg	S	Zn	Cu	Mn	Fe	B
	g·kg^–1^						mg·kg^–1^				
control	43.1	7.7	45.4	5.7	3.8	2.3	43.3	5.5	133.4	154.8	35.0
25%	34.0	7.2	44.9	7.9	4.1	1.9	34.1	5.2	127.0	168.4	27.4
50%	35.2	7.2	37.8	5.5	3.4	2.3	41.6	5.1	120.0	104.5	26.6
75%	29.0	9.1	45.4	8.1	5.9	2.9	61.6	7.3	225.5	163.8	25.8
100%	36.5	7.5	42.5	5.7	4.5	2.0	51.6	5.3	172.3	127.1	18.1

Mungkunkamchao et al.^29^ observed that tomato plants reduced potential nutritional deficiencies when sprayed with eucalyptus pyrolysis oil. Rice straw bio-oil showed promising results in rice crops’ grain productivity, panicles, and grain filling. Higher grain yield was observed due to greater tillering, which was also observed in the treatment with the liquid fraction of pyrolysis. These results, associated with those obtained in the present study, show that pyrolysis oil, regardless of the raw material used, has potential for application in agriculture in different crops, with little or no negative impact on their development.^30^

Pyrolysis oil, when used as a pH regulator in hydroponic lettuce cultivation (since it has a pH close to 4.0 for most biomasses), did not harm plant development and maintained the pH at the desired levels. Thus, this material can be a viable alternative to nitric acid, which is highly corrosive and dangerous and is commonly used to adjust the pH in hydroponic systems. Furthermore, these results align with those of the present study in that pyrolysis oil does not cause significant adverse effects, even when applied to crops considered sensitive.^31^

In a study where manure and grass, organic materials widely used in agriculture and home gardens, were applied, an increase in Zn levels was observed in lettuce leaves, as in this study at the highest doses (75% and 100% v/v).^32^ Such a result indicates that not only inorganic material but also organic material containing metals can cause an increase in the levels of macro- and micronutrients in plants. However, the necessary quantities vary, depending on the levels present in each type of material.

Regarding nutrient absorption, the values did not follow a progressive linear increase with increasing concentration. However, there was also no decrease, which shows that pyrolysis oil did not harm plant development, concerning nutrient content. This was also observed by Zhang et al.^25^ in blueberry fruits, which showed no difference in the content of the nutrients analyzed.

The levels of residual nutrients of fertilizers alone and in combination with pyrolysis oil in watermelon plants were evaluated, and it was possible to observe that the treatments with fertilizers presented higher values. However, they did not differ statistically from those containing bio-oil. This behavior was like that observed in the present study, where the levels of micro- and macronutrients did not change with the treatment with the material.^26^

As the type of material used for pyrolysis (municipal solid waste) may contain a wide variety of materials, possibly including batteries, paint packaging, insecticides, cleaning products, lamps, and aerosols, which are disposed of incorrectly, it is imperative to check for the presence of heavy metals in pyrolysis oil obtained from possibly contaminated raw materials.^33^

During the pyrolysis process, heavy metals present in the raw material can be transferred to different products through chemical and physical reactions caused by heat.^34^ When a material containing heavy metals is destined for pyrolysis, the main objective is to retain the highest level of contaminants in solid products and the lowest in volatiles.^35^

High temperatures facilitate the evaporation of contaminants, from the solid fraction to the volatile ones.^36^ Yuan et al.^37^ evaluated the presence of heavy metals in sewage sludge pyrolysis oil. They found that the material contained high levels of Cd, Zn, and Ni, but they became more worrisome at temperatures above 800 °C. This is due to the decrease in volatile components due to high temperatures, while at lower temperatures, heavy metals can migrate to gas or liquid fractions.^38^ Materials from metal-rich raw materials must be pretreated or improved before use.

The parameters adopted to execute the pyrolysis process also influence the amount of heavy metals in the liquid fraction. Other studies have reported that pyrolysis oils produced through fast pyrolysis contained insignificant amounts of heavy metals without any pre- or post-treatment of the product obtained from the thermochemical process.^39,40^

Given this problem, the levels of heavy metals in the lettuce treated with MSW pyrolysis oil were evaluated, and the results are compiled in Table 5.

**Table 5:** Heavy Metal Levels Found in Lettuce Samples Treated with Increasing Municipal Solid Waste Pyrolysis Oil Doses^a^

heavy metal (mg·kg^–1^)											
	As	Cd	Pb	Al	Ba	Co	Cr	Sn	Hg	Mo	No
control	<LQ	0.076	0.061	204.21	6.24	0.107	1.79	0.055	<LQ	3.34	2.02
25%	<LQ	0.099	0.049	138.15	5.40	0.081	0.53	<LQ	<LQ	5.478	0.71
50%	<LQ	0.102	0.091	207.80	8.64	0.051	0.57	<LQ	<LQ	3.149	0.72
75%	<LQ	0.146	0.072	361.42	5.43	0.117	0.95	0.04	<LQ	1.989	1.93
100%	<LQ	0.166	0.154	198.83	6.35	0.278	3.02	0.073	<LQ	2.419	3.12

a <LQ—Less than the quantification limit of the method (LQ_As_ = 0.0272 mg·kg^–1^, LQ_Sn_ = 0.0590 mg·kg^–1^, and LQ_Hg_ = 0.0046 mg·kg^–1^).

MSW pyrolysis oil was produced at a temperature of 450 °C. Zhong et al.^41^ analyzed the heavy metal contents in the pyrolysis oil of *Sedum plumbizincicola*.^42^ They concluded that Pb and Cd were volatilized mainly at reactor temperatures higher than 450 °C and were condensed into the oil, which can be observed in the present work, where trim levels of these metals were observed in the leaf tissue of lettuce plants.

Table 5 shows that in some cases, the control had higher values of heavy metals than the treatments that contained oil. These heavy metals can be incorporated into the biomass during its normal growth in an unintentional way, as well as through fertilizer applications, seed treatments, and industrial activities in nearby locations.^38^

Nationally, ANVISA defines the concentration limits of inorganic contaminants in food in Brazil. These values are established based on studies carried out by ANVISA itself as well as by the Food and Agriculture Organization of the United Nations (FAO) and the World Health Organization (WHO). At a global level, the values are defined by the World Health Organization (WHO).^43^

Table 6 presents a food safety analysis based on the results found based on the heavy metal levels and the estimated consumption of 130 g of lettuce submitted to the experiment. For the calculation, an adult weighing 72 kg was considered, like the study by Ribeiro et al.^44^

**Table 6:** Survey of the Percentage of Daily Consumption of Heavy Metals through Lettuce Treated with Pyrolysis Oil from Municipal Solid Waste, Considering a Consumption Quota of 130 g of Fresh Plant per Day^a^

treatment	daily intake (μg)					total daily intake (% relative to maximum dose)				
	As	Cd	Cu	Hg	Pb	As	Cd	Cu	Hg	Pb
control	—	9.8	715	—	7.9	—	16.4	23.8	—	3.7
25% v/v	—	31.1	676	—	6.4	—	51.9	22.5	—	3.0
50% v/v	—	13.2	663	—	11.8	—	22.1	22.1	—	5.6
75% v/v	—	18.9	949	—	9.3	—	31.6	31.6	—	4.4
100% v/v	—	21.5	728	—	20.0	—	35.9	24.2	—	9.5

a Maximum permitted intake according to the WHO:^53^ As – 2 μg·day^–1^; Cd – 60 μg·day^–1^; Cu – 3000 μg·day^–1^; Hg – 42 μg·day^–1^; Pb – 210 μg·day^–1^. The dash ′-′ indicates that the element was below the method’s detection limit (LQ_As_ = 0.0272 mg·kg^–1^ and LQ_Hg_ 0.0046 mg·kg^–1^) and could not be quantified.

The metals that presented the highest percentages of daily intake in 130 g of cultivated vegetables were Cd and Cu. It is interesting to note that consuming food contaminated with heavy metals can cause serious health risks, such as neurological, genotoxic, metagenomic, carcinogenic, and respiratory problems.^45^

Normative Instruction No. 160, of July 1, 2022, from ANVISA,^46^ defined maximum tolerable limits for contaminants in food and the values for some heavy metals. The values indicate that the application of pyrolysis oil from urban waste did not cause a significant increase in the content of heavy metals in the sample since they remained within the limits permitted by law. Even so, care regarding the use of this product in the production process cannot be ignored since lettuce is one of the main vegetables consumed by the Brazilian population, and its ingestion with excessive levels of contaminants can increase health risks.

Sampaio et al.^47^ characterized qualitatively and quantitatively the presence of heavy metals in lettuce when fertilized with urban waste compost. The authors reported that Cu levels increased in leaf tissue, while Ni and Cd levels decreased. It was noted that the accumulation behavior of the metals differed depending on the application method.

The levels of phenolic and flavonoid compounds evaluated for treatments with pyrolysis oil from urban solid waste did not differ statistically from those of the control, whose values can be seen in Table 7.

**Table 7:** Phenolic and Flavonoid Contents in Lettuce Treated with Increasing Doses of Municipal Solid Waste Pyrolysis Oil^a^

treatment	phenolics (mg·100 g^–1^)	flavonoids (mg·100 g^–1^)
control	80.2 ± 12.0^NS^	114.4 ± 14.8^NS^
25%	88.1 ± 11.6	111.5 ± 20.2
50%	98.0 ± 7.8	130.6 ± 30.7
75%	94.0 ± 13.3	100.2 ± 25.7
100%	93.5 ± 13.6	129.9 ± 15.1
coefficient of variation (%)	13.08	18.91

a ^NS^—not significant by the ANOVA at a 5% error probability.

As shown in Table 7, the levels did not decrease, demonstrating that the application of pyrolysis oil from urban solid waste did not cause harm to the plants or stimulate the production of secondary metabolites, which would indicate a possible stressor effect on the plants. According to data in the literature, bio-oil from wood waste did not significantly affect the levels of phenolic compounds and flavonoids in blueberries.^25^

Bioactive compounds, including phenolic and flavonoid compounds, indicate beneficial effects due to antioxidant and antiproliferative activity, which are directly linked to a reduction in the development of atherosclerosis and cancer.^5^ At an agronomic level, the higher the content of bioactive compounds present in the plant, the greater its resistance to sources of stress, such as drought, excessive sunlight, and attacks by predators and pathogens, for example.

Tomato fruits (Solanum lycopersicum L.) were treated with eucalyptus pyrolysis oil and combined with a fermented bioextract. According to the authors, tomatoes showed increased soluble solids content only in the foliar application of the combination of treatments. This demonstrates again that pyrolysis oil has promising potential when associated with a product already on the market.^29^

The pyrolysis process is currently seen as a sustainable means of managing nonrecyclable waste and biomass that is not reused and discarded. In addition, pyrolysis byproducts are under investigation to verify their agricultural suitability and potential use to act together with or replace synthetic products, causing less environmental and human health impact.^48^

Several studies have addressed the characterization of pyrolysis oil from different raw materials. The different pyrolysis oils are characterized by presenting substantial amounts of esters, alcohols, acids, sugars, and phenols. However, it is important to note that the composition is quite variable, depending on the type of biomass and the pyrolysis process employed. Despite the intrinsic variability, there is evidence that this type of material has great potential in agriculture.^49^

Brazil is the world’s largest consumer of agricultural inputs (in terms of kilograms of inputs per hectare of cultivated area). Considering that agriculture contributes most to the Brazilian economy, using fertilizers and phytosanitary products can cause damage to the environment and economic dependence.^50^ The price of inputs was responsible for the increase in the production cost of Brazilian agriculture in 2022, with some products having increased by up to 100% of their value.^51^ The historical series between 2010 and 2021 shows a 53.21% increase in the commercialization of inputs, exceeding 700 thousand tons of active ingredient in the last harvest recorded.^52^ This demonstrates the need to search for new alternative inputs, which can combine the need to increase agricultural production with the reduction of waste generation and material destined for disposal rather than reuse or as raw material for some production processes.

It is important to point out that lettuce was used in this study because it is a ‘model plant’ for toxicity and growth tests. The pyrolysis oil of MSW and other kinds of feedstocks must be properly assessed before its integration into the vegetable productive chain. Nevertheless, the present study showed the potential use of MSW pyrolysis oil in nonfood crops, such as species for forestry and industrial uses.

## 4 Conclusion

The pyrolysis oil presented a very heterogeneous characterization with a great diversity of compounds, requiring a quantitative analysis of the compounds for a more practical discussion. However, it is possible to highlight the presence of phenols in the compound. Germination control of lettuce plants was satisfactory, where increasing the concentration led to proportional inhibition of seed germination. Higher concentrations may lead to pre-emergent herbicidal effects on plants. Plants treated with pyrolysis oil from urban solid waste did not risk contamination by heavy metals since the levels did not exceed the maximum limits recommended by regulations. It is also important to note that pyrolysis oil did not cause phytotoxicity on lettuce when applied by an aspersion. Applying the liquid fraction of pyrolysis did not significantly alter the nutrient levels in the plants. However, studies have shown that combining it with other products already on the market could become an alternative. Regarding the levels of phenolic compounds and flavonoids, there was no significant difference between the control and other treatments, and it was possible to verify that their use in the crop does not have a stressful effect on lettuce. Pyrolysis is a viable alternative for waste disposal, regardless of the raw material. However, the search for the use of the products generated in the process is essential, as long as it is done safely and efficiently. Making part of this material viable as a benefit for agricultural production is a combination of important links: the environment and agriculture. Further studies are recommended to assess the potential of using MSW pyrolysis oil in other crops, both food and industrial, and under different cultivation conditions.
